# Executive Functioning, Emotion Regulation, and Negative Emotion in Suicidal Older Adults

**DOI:** 10.1093/geroni/igab046.2648

**Published:** 2021-12-17

**Authors:** Rachel Waldman, Brian Liles, Dimitris Kiosses, Richard Zweig

**Affiliations:** 1 Weill Cornell Medicine, New York, New York, United States; 2 Weill Cornell Medicine, White Plains, New York, United States; 3 Weill-Cornell Medicine, White Plains, New York, United States; 4 Yeshiva University, Bronx, New York, United States

## Abstract

Deficits in executive functioning, emotion regulation, and negative emotion have all been linked to suicidality. Yet, the complex interactions between these three factors and their relationships to suicidal behavior in older adults remain unclear. Participants (N = 39) were depressed middle and older adult (M = 62.0, SD = 9.41) inpatients with recent suicidal attempt or ideation, without psychotic depression or moderate or greater cognitive impairment (DRS>90). Participants were administered measures of executive functioning (Stroop and COWAT), emotion regulation (ERQ Suppression and Reappraisal; RRS-Brooding; UPPS- Premediation Scale), and negative emotion (PANAS-X), in addition to measures of depression (MADRS) and suicidality (C-SSRS). Results indicated that executive functioning was not significantly related to emotion regulation or negative affect, but measures of emotion regulation were related to negative emotion and frequency of suicidal ideation in bivariate analyses. Lower ERQ reappraisal tended to be associated with negative emotion (ß = -.392, p = .067) in multivariate analyses. Although comparisons were non-significant, effect sizes revealed that those who experienced daily suicidal ideation (C-SSRS) had lower reappraisal and higher brooding scores (Cohen’s d = 1.014 - 1.456), as well as higher executive functioning (Stroop Color-Word trial) and overall cognition (DRS) scores (Cohen’s d = 0.625 – 0.792) than less frequent ideators. Findings suggest that older inpatients with frequent suicidal ideation have poorer emotion regulation but may have more intact cognition and executive functioning than those with less frequent suicidal ideation.

